# Inhibition of Notch pathway prevents osteosarcoma growth by cell cycle regulation

**DOI:** 10.1038/sj.bjc.6605060

**Published:** 2009-05-19

**Authors:** M Tanaka, T Setoguchi, M Hirotsu, H Gao, H Sasaki, Y Matsunoshita, S Komiya

**Affiliations:** 1 Department of Orthopaedic Surgery, Graduate School of Medical and Dental Sciences, Kagoshima University, 8-35-1 Sakuragaoka, Kagoshima 890-8520, Japan

**Keywords:** osteosarcoma, Notch, cell cycle, *γ*-secretase inhibitor, CBF1

## Abstract

The study shows constitutive activation of the Notch pathway in various types of malignancies. However, it remains unclear how the Notch pathway is involved in the pathogenesis of osteosarcoma. We investigated the expression of the Notch pathway molecules in osteosarcoma biopsy specimens and examined the effect of *Notch* pathway inhibition. Real-time PCR revealed overexpression of *Notch2, Jagged1, HEY1*, and *HEY2*. On the other hand, *Notch1* and *DLL1* were downregulated in biopsy specimens. Notch pathway inhibition using *γ*-secretase inhibitor and *CBF1* siRNA slowed the growth of osteosarcomas *in vitro*. In addition, *γ*-secretase inhibitor-treated xenograft models exhibited significantly slower osteosarcoma growth. Cell cycle analysis revealed that *γ*-secretase inhibitor promoted G1 arrest. Real-time PCR and western blot revealed that *γ*-secretase inhibitor reduced the expression of accelerators of the cell cycle, including cyclin D1, cyclin E1, E2, and SKP2. On the other hand, p21^cip1^ protein, a cell cycle suppressor, was upregulated by *γ*-secretase inhibitor treatment. These findings suggest that inhibition of Notch pathway suppresses osteosarcoma growth by regulation of cell cycle regulator expression and that the inactivation of the Notch pathway may be a useful approach to the treatment of patients with osteosarcoma.

Osteosarcoma is the most common primary bone cancer occurring mainly in children (Gibbs *et al*, 2002). After initial diagnosis is made by biopsy, treatment consists of preoperative chemotherapy, followed by definitive surgery and postoperative chemotherapy. Survival has improved over the past several decades. Indeed, patients with non-metastatic disease have a 70% chance of long-term survival. Unfortunately, patients with metastatic disease at diagnosis or those who have recurrent disease have a poor prognosis, with only 20% surviving at 5 years, indicating that new therapeutic options for them need to be actively explored. In cancer cells, dysregulation of cell division and apoptotic processes contribute to drug resistance and to metastatic potential (Igney and Krammer, 2002; Lafleur *et al*, 2004). It is reported that the cell cycle regulatory pathway centred around pRb is frequently somatically inactivated in osteosarcomas (Horowitz *et al*, 1990). Although such dysregulation may represent a potent source of new therapeutic targets, the molecular mechanisms that regulate osteosarcoma cell proliferation are largely unknown.

Members of the Notch family are highly conserved transmembrane receptors that influence the proliferation and apoptosis of diverse types of cells in a variety of organisms (Artavanis-Tsakonas *et al*, 1999). Activation of Notch signalling requires binding of its ligands (Jagged, DLL), followed by proteolytic release of the Notch intracellular domain (NIC) and its translocation to the nucleus (De Strooper *et al*, 1999). Notch intracellular domain interacts with CSL transcription factors (CBF1/RBP-J*κ*, Su(H), Lag-1) and converts them from repressors to activators, promoting transcription of downstream genes involved in various differentiation programmes (Davis and Turner, 2001). Recent studies have also shown constitutive activation of the Notch pathway in various types of malignancies. The oncogenic potential of Notch has been reported in human T-cell acute lymphoblastic leukaemia (Grabher *et al*, 2006), non-small cell lung cancer (Dang *et al*, 2000), ovarian carcinomas (Park *et al*, 2006), and pancreatic cancer (Miyamoto *et al*, 2003). Although, it was recently reported that DLL1, Notch1, Notch2, and HES1 are expressed in osteosarcoma cell lines, whether expression of Notch signalling molecules in osteosarcoma patient specimens is aberrant has not been clarified.

To explore the involvement of aberrant Notch signalling in the pathogenesis of osteosarcoma, we investigated the expression of the Notch pathway molecules in osteosarcoma patient specimens and examined the effects of Notch pathway inhibition by *γ*-secretase inhibitor (GSI) and CBF1 siRNA. We found that *Notch2, Jagged1, HEY1*, and *HEY2* were upregulated in the osteosarcoma biopsy specimens. On the other hand, *Notch1* and *DLL1* were downregulated in biopsy specimens. In addition, Notch pathway inhibition prevented the growth of osteosarcoma *in vitro* and *in vivo* by cell cycle regulation.

## Materials and methods

### Cell culture

Human osteosarcoma cell lines, HOS, 143B, Saos-2, and U2OS cells were purchased from the American Type Culture Collection (ATCC). Cells were grown in Dulbecco's modified Eagle's medium (DMEM) supplemented with 10% FBS, penicillin (100 U ml^−1^), and streptomycin (100 *μ*g ml^−1^). Human osteoblast cells (NHOst) were purchased from Sanko Junyaku (Tokyo, Japan). Cells were cultured with OBM (Cambrex, East Rutherford, NJ, USA) or DMEM supplemented with 10% FBS. All cells were grown in a humidified atmosphere containing 5% CO_2_ at 37°C.

### Patient specimens

All osteosarcoma biopsy specimens were obtained from primary lesions. Biopsy was carried out before chemo- or radiotherapy to make the diagnosis. Normal bone tissue was obtained from femur during total hip arthroplasty.

### Real-time PCR (RT–PCR)

For real-time PCR (RT-PCR), total RNA was DNase-treated and reverse transcribed using oligo(dT) primers as described by the manufacturer (Invitrogen, Carlsbad, CA, USA). Reactions were run using SYBR Green (BIO-RAD, Hercules, CA, USA) on a MiniOpticon machine (BIO-RAD). The comparative *C*_t_ (ΔΔ*C*_t_) method was used to determine fold change in expression using *β*II-microglobulin. Each sample was run minimally at three concentrations in triplicate. All primer sets amplified 100 to 200 bp fragments. The primer sequences used were as follows: for Notch1: 5-GTGACTGCTCCCTCAACTTCAAT-3, 5-CTGTCACAGTGGCCGTCACT-3; Notch2: 5-AAAAATGGGGCCAACCGAGAC-3, 5-TTCATCCAGAAGGCGCACAA-3; Jagged1: 5-CGGGATTTGGTTAATGGTTATC-3, 5-ATAGTCACTGGCACGGTTGTAGCAC-3; DLL1: 5-CCTACTGCACAGAGCCGATCT-3, 5-GCAGGTGGCTCCATTCTTGC-3; HES1: 5-AGGCGGACATTCTGGAAATG-3, 5-CGGTACTTCCCCAGCACACTT-3; HEY1: 5-CGAGGTGGAGAAGGAGAGTG-3, 5-CTGGGTACCAGCCTTCTCAG-3; HEY2: 5-GAACAATTACTCGGGGCAAA-3, 5-TCAAAAGCAGTTGGCACAAG-3; CBF1: 5-CGCATTATTGGATGCAGATG-3, 5-CAGGAAGCGCCATCATTTAT-3; cyclin D1: 5-ACAAACAGATCATCCGCAAACAC-3, 5-TGTTGGGGCTCCTCAGGTTC-3; cyclin E1: 5-CCACACCTGACAAAGAAGATGATGAC-3, 5-GAGCCTCTGGATGGTGCAATAAT-3; cyclin E2: 5-TGTTGGCCACCTGTATTATCTGG-3; 5-ATCTGGAGAAATCACTTGTTCCTATTTC-3; SKP2: 5-TGGGAATCTTTTCCTGTCTG-3, 5-GAACACTGAGACAGTATGCC-3; c-Myc: 5-GCCACGTCTCCACACATCAG-3, 5-TGGTGCATTTTCGGTTGTTG-3; *β*II-microglobulin: 5-TCAATGTCGGATGGATGAAA-3, 5-GTGCTCGCGCTACTCTCTCT-3.

### MTT assay

Cells were incubated with substrate with MTT (3-(4,5-dimethylthiazol-2-yl)-2,5-diphenyltetrazolium bromide) for 4 h, and washed with PBS and lysed to release formazan from cells. Then cells were analysed in a Safire microplate reader (BIO-RAD) at 562 nm. *γ*-Secretase inhibitor X was used for MTT assay.

*CBF1* siRNA was purchased from Santa Cruz (Santa Cruz, CA, USA). Lipofection of siRNA was carried out every other day as recommended in the supplier's protocol using FuGENE 6 (Roche, Basel, Switzerland).

### Cell death detection

Cell death detection ELISA^plus^ (Roche) was used to detect cell death as per manufacturer's protocol. Briefly, cells were incubated with different concentrations of camptothecin (CAM) for 4 h at 37°C. Before and after lysis, cells were centrifuged and the supernatant was analysed.

### Immunohistochemistry

The following primary antibodies were used: anti-HES1 (diluted 1 : 200; Chemicon, Temecula, CA, USA) and ki67 (Zymed Laboratories, San Francisco, CA, USA). The following secondary antibodies were used; fluorescein rhodamine-conjugated donkey antirabbit IgG antibody (diluted 1 : 200; Chemicon). The cells were counterstained with Hoechst 33258 (Molecular Probes, Carlsbad, CA, USA) to identify nuclei. Immunohistochemistry with each second antibody alone without primary antibody was carried out as a control.

### Western blot

Cells were lysed using NP40 lysis buffer (0.5% NP40, 10 mM Tris-HCl (pH 7.4), 150 mM NaCl, 3 mM pAPMSF (Wako Chemicals, Kanagawa, Japan), 5 mg ml^−1^ aprotinine (Sigma, St Louis, MO, USA), 2 mM sodium orthovanadate (Wako Chemicals), and 5 mM EDTA). Lysates were subjected to SDS–PAGE and subsequent immunoblotting with antibodies to actin, cyclin D1, E1, E2, p21, SKP2, pRb, c-Myc (Santa Cruz), and Notch2-inter cellular domain (Abcam, Cambridge, UK). Detection was carried out using the ECL detection system (Amersham, Chalfont St Giles, UK).

### Animal experiments

In all, 143B cells (1 × 10^6^) were mixed with collagen gel in a 1 : 1 volume, and inoculated subcutaneously in 5-week-old nude mice. The mice were randomly assigned to receive either GSI XX (10 *μ*g kg^−1^; CALBIOCHEM, Basel, Switzerland) or an equal volume of physiological saline solution (control). *γ*-Secretase inhibitor XX and saline solution were administered by intraperitoneal injection. The treatment with GSI XX was initiated 1 week after tumour inoculation when the tumours had grown to visible size. Tumour size was measured with calipers weekly, and tumour volume was calculated using the formula LW^2^/2 (with L and W representing the length and width of tumours). All experimental procedures were carried out in compliance with the guiding principles for the Care and Use of Animals described in the *American Journal of Physiology* and with the Guidelines established by the Institute of Laboratory Animal Sciences, Faculty of Medicine, Kagoshima University. All efforts were made to minimise animal suffering, to reduce the number of animals used, and to utilise possible alternatives to *in vivo* techniques.

### Cell cycle analysis

Cell cycle analysis was committed and carried out by Reprocell (Tokyo, Japan). At 48 h after GSI X treatments, cells were collected by trypsinisation and washed with DPBS. Cells were fixed in 70% (v/v) ethanol at 4°C, washed with PBS, and resuspended with 500 *μ*l of staining solution (PBS pH 7.4, 100 *μ*g ml^−1^ DNase-free RNase, 1 mg ml^−1^ propidium iodide). Cells were then analysed by flow cytometry using an FACS Vantage SE (Becton Dickinson, Franklin Lakes, NJ, USA). Data were gated using pulse width and pulse area to exclude doublets, and the percent of cells present in each phase of the cell cycle was calculated using FlowJo software (Tree Star, Ashland, OR, USA).

### Statistics

Each sample was analysed in triplicate, and experiments were repeated three times. In all figures, error bars are s.d. All statistical analyses were carried out using Microsoft Office Excel (Microsoft, Albuquerque, New Mexico, USA) and STASTISCA (StatSoft, Tulsa, OK, USA). Differences between mean values were evaluated by the unpaired *t*-test, and differences in frequencies were evaluated by Fisher's exact test. Differences were considered significant at *P*<0.05.

## Results

### *Notch2, Jagged1, HEY1,* and *HEY2* are overexpressed in osteosarcoma patient specimens

**Figure 1:**
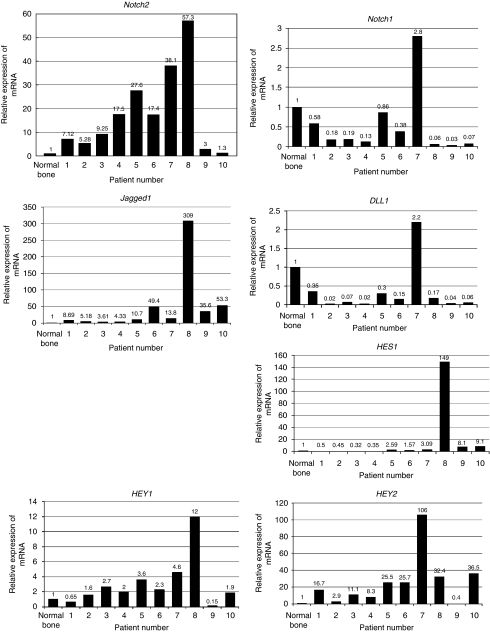
Notch pathway molecules are overexpressed in osteosarcoma patient specimens. Total RNA extracted from osteosarcoma biopsy specimens was used for real-time PCR. Ten of ten human biopsy specimens of osteosarcoma increased Notch2 1.3–57.3-fold. Notch1 was decreased 0.03–0.86-fold in 9 of 10 biopsy specimens. Jagged1 was upregulated 3.6–309-fold in 10 of 10 biopsy specimens. In 9 of 10 human biopsy specimens, DLL1 was decreased 0.02–0.35-fold. HES1 was upregulated in 6 of 10 and downregulated in 4 of 10 biopsy specimens. HEY1 was upregulated 1.6–12-fold in 8 of 10 biopsy specimens. HEY2 was upregulated 2.9–106-fold in 9 of 10 biopsy specimens. The comparative *C*_t_ (ΔΔ*C*_t_) method was used to determine fold change in expression using *β*II-microglobulin. Each sample was run minimally at three concentrations in triplicate.

Real-time PCR was carried out to examine the gene expression of Notch pathway molecules. Real-time PCR revealed that 10 of 10 human biopsy specimens of osteosarcoma increased *Notch2* 1.3–57.3-fold (Figure 1). On the other hand, *Notch1* was decreased 0.03–0.86-fold in 9 of 10 human biopsy specimens (Figure 1). To further examine Notch pathway molecules expression, we carried out RT-PCR for Notch ligands and Notch target genes. It was reported that Jagged1 and DLL1 are Notch ligands (Bettenhausen *et al*, 1995; Oda *et al*, 1997). *Jagged1* was upregulated 3.6–309-fold in 10 of 10 human biopsy specimens of osteosarcoma (Figure 1). On the other hand, *DLL1* was decreased 0.02–0.35-fold in 9 of 10 human biopsy specimens (Figure 1). It was reported that *HES* and *HEY* are Notch target genes (Jarriault *et al*, 1995; Maier and Gessler, 2000). *HES1* was upregulated in 6 of 10 and downregulated in 4 of 10 biopsy specimens (Figure 1). *HEY1* was upregulated 1.6–12-fold in 8 of 10 human biopsy specimens (Figure 1). *HEY2* was upregulated 2.9–106-fold in 9 of 10 human biopsy specimens (Figure 1). Immunohistochemical examination revealed that HES1 was accumulated in the nuclei of human osteosarcoma samples (Supplementary data A). These findings suggest that the Notch signalling pathway is activated in human osteosarcomas.

### Inhibition of Notch pathway prevents osteosarcoma growth *in vitro* and *in vivo*

**Figure 2:**
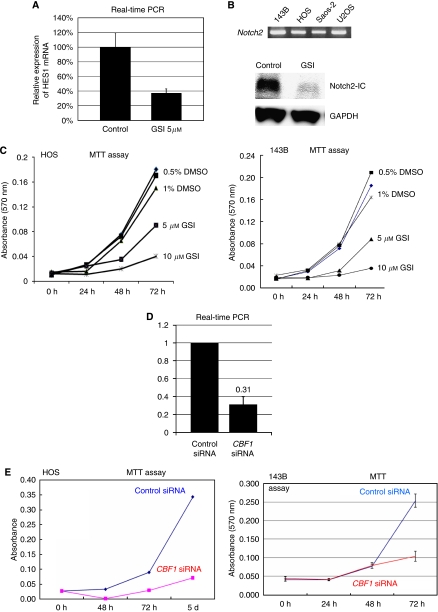
Inhibition of Notch pathway prevents proliferation of osteosarcoma *in vitro*. Real-time PCR revealed four human osteosarcoma cell lines that express *Notch2* (**B**). We carried out RT–PCR to determine which concentration of *γ*-Secretase inhibitor (GSI) X effectively inhibited Notch activity in osteosarcoma cells, and then measured the expression of the Notch pathway target *HES1*. *γ*-Secretase inhibitor X at 5 *μ*M reduced mRNA levels of HES1 in 143B cells more than 60% (error bar means s.d.) (**A**). Western blot analysis showed that 5 *μ*M GSI treatment reduced Notch2 intercellular domain (**B**). Growth of viable HOS and 143B cells over 3 days was slowed in dose-dependent fashion by GSI X (**C**). Real-time PCR revealed that siRNA knocked down *CBF1* mRNA about 69% (**D**). Growth of HOS and 143B cells was slowed by *CBF1* siRNA (**E**).

**Figure 3:**
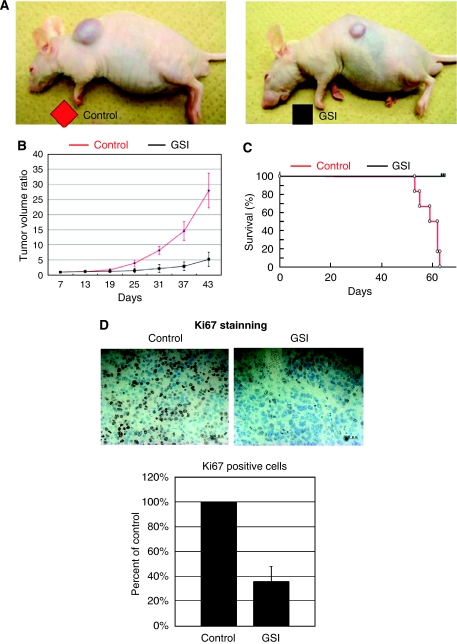
Notch pathway inhibition blocks osteosarcoma xenograft growth *in vivo* and prolongs survival. In all, 143B cells (1 × 10^6^) were inoculated subcutaneously. Established 143B tumours were measured and then injected with *γ*-secretase inhibitor (GSI) or DMSO intraperitoneally. The tumour volume at day 7 was set at 1, and tumour volumes at subsequent time points were calculated. *γ*-Secretase inhibitor significantly inhibited tumour growth at day 31 compared with DMSO. The following decreases in tumour volume were observed in GSI compared with DMSO treatment: day 25: 37.9%; day 31: 26.3%; day 37: 19.7%; and day 43: 18.6% (**A** and **B**). Kaplan–Meier survival curves from GSI treatment groups (black) and DMSO control (red). Kaplan–Meier analysis showed that GSI administration conferred a significant survival benefit (**C**; *n*=6, *P*<0.05). Immunohistochemical examination of ki67 was carried out in xenograft tumours. Ki67 staining revealed that proliferation of osteosarcoma cells was decreased by GSI treatment. The number of Ki67-positive cells was decreased to 36% of control revel by GSI administration at day 35 (**D**; error bar means s.d.).

To determine whether Notch pathway activation is required for osteosarcoma cell growth and survival, we used GSI X, a pharmacological agent known to effectively block Notch activation by inhibiting the proteolysis and translocation of NIC to the nucleus. We carried out RT-PCR to determine which concentration of GSI X effectively inhibited Notch activity in osteosarcoma cells, and then measured the expression of the Notch pathway target *HES1*. In 143B cell, GSI X at 5 *μ*M reduced mRNA levels of *HES1* in 143B cells more than 60% (Figure 2A). As GSI-18 was used to prevent glioma cell growth at 2–10 *μ*M (Fan *et al*, 2006), we decided that 5 *μ*M was appropriate concentration for osteosarcoma. In addition, western blot showed that 5 *μ*M GSI X decreased the Notch2-inter cellular domain (Figure 2B). These data suggest that Notch signalling is blocked as expected with GSI. We then assessed the tumour growth *in vitro* under GSI treatment. MTT assay revealed that GSI treatment slowed the growth of HOS and 143B in dose-dependent fashion, suggesting that this concentration is sufficient to induce the antitumour effect mediated by GSI (Figure 2C). On the other hand, cell death detection assay revealed that GSI did not affect cell death (Supplementary data B). To confirm the effects of Notch pathway inhibition, we examined those of *CBF1* siRNA. When NIC translocates to the nucleus, it binds CBF1 and activates transcription of target genes. Real-time PCR revealed that siRNA effectively knocked down *CBF1* mRNA (Figure 2D). MTT assay revealed that viable cell mass was reduced by *CBF1* siRNA in HOS and 143B (Figure 2E). We next examined the effects of Notch pathway blockade on tumour formation *in vivo*. Nude mice were inoculated with 143B osteosarcoma cells intradermally, and palpable tumours formed in 7 days. The mice were then injected intraperitoneally in GSI or DMSO as a control as reported earlier (van Es *et al*, 2005). The injections were repeated every other day. Results showed significant inhibition of tumour growth in the GSI-treated *vs* DMSO-treated control group. All of the 6 GSI-treated tumours exhibited significantly slower growth than DMSO-treated tumours (Figure 3A and B). Kaplan–Meier analysis showed that GSI administration conferred a significant survival benefit (Figure 3C). Histological analysis indicated that GSI induced growth arrest. The control tumours exhibited a number of cells positive for Ki67, a marker of cell proliferation. In contrast, GSI-treated tumours exhibited little evidence of proliferation, as evidenced by lack of Ki67 positivity. The number of Ki67-positive cells was decreased to 36% of control level by GSI administration (Figure 3D).

### Notch pathway regulates osteosarcoma cell cycle

**Figure 4:**
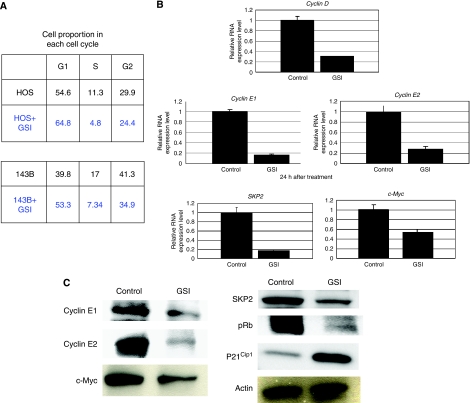
Notch pathway inhibition promotes G1 arrest. HOS and143B cells were treated with 5 *μ*M GSI. After 48-h treatment, cells were collected and subjected to cell cycle analysis. When HOS cells were cultured without GSI, 54.6% of cells were in G1 phase. On the other hand, when cultured with GSI, 64.8% of cells were in G1 phase. In the case of 143B cells cultured without GSI, 39.8% of cells were in G1 phase, whereas 53.3% of cells were in G1 phase when treated with GSI (**A**). Real-time PCR was carried out to quantify mRNAs of cell cycle-related genes. A 24-h treatment with GSI reduced the levels of *cyclin D, cyclin E1, E2, SKP2*, and *c-Myc* transcription (error bar means s.d.; **B**). Western blot analysis of the levels of cell cycle-related genes. 48 h treatment with GSI reduced the levels of expression of cyclin E1, cyclin E2, c-Myc, pRb, and SKP2 proteins. Expression of P21^cip1^ protein was upregulated by GSI treatment. The experiment was triplicate with similar results (**C**; GSI: 5 *μ*M).

We examined cell cycle characteristics by flow cytometry. When HOS cells were cultured without GSI, 54.6% of cells were in G1 phase. On the other hand, when cultured with GSI, 64.8% of cells were in G1 phase. In the case of 143B cells cultured without GSI, 39.8% of cells were in G1 phase, whereas 53.3% of cells were in G1 phase when treated with GSI (Figure 4A). These findings suggested that GSI promoted G1 arrest. We then examined the transcription of genes related to the cell cycle. Real-time PCR revealed that GSI prevented the transcription of accelerators of the cell cycle, including *cyclin D*, *cyclin E1*, *cyclin E2, SKP2*, and *c-Myc* (Figure 4B). In mammalian cells, cyclin D, cyclin E, and p21^cip1^ are short-lived proteins that are controlled by ubiquitin-dependent proteolysis. Western blot examination showed that GSI reduced the levels of expression of cyclin E1, cyclin E2, c-Myc, p-Rb, and SKP2 proteins. We next examined the expression of p21^cip1^, and found that p21^cip1^ protein was upregulated by GSI treatment (Figure 4C). These findings suggested that GSI promoted G1 arrest by inhibition of G1-S phase progression.

## Discussion

Deregulation of Notch signalling is implicated in the development of various cancers, and Notch blockade appears to affect the survival and proliferation of multiple types of cancer (Radtke and Raj, 2003; Politi *et al*, 2004; Weng and Aster, 2004). For example, *Notch* is activated by translocation or mutation in more than half of T-cell acute lymphoblastic leukaemias, and anti-Notch treatments have been shown to slow the growth of acute lymphoblastic leukaemia growth *in vitro* (Weng *et al*, 2004). In addition, combining GSIs and anticancer drugs improve anticancer effect (Meng *et al*, 2009; Real *et al*, 2009). In this study, we found that *Notch2, Jagged1, HEY1*, and *HEY2* were overexpressed in human osteosarcoma specimens. On the other hand, expression of *Notch1* and *DLL1* was downregulated in biopsy specimens. Recently, Zhang *et al* (2008) reported that osteosarcoma cell lines, including OS187, COL, SAOS2, and LM7, expressed Notch-related molecules. Real-time PCR revealed that *Notch1* expression was upregulated in three of four cell lines. *Notch2* expression was upregulated in two of four cell lines, and *HES1* expression was upregulated in two of four cell lines. These results suggested that expression of Notch-related genes differs markedly among cell lines and patient specimens. Zhang *et al* (2008) carried out RT–PCR for two osteosarcoma patient specimens and found that both expressed *Notch1, Notch2, Notch4, HES1*, and *HERP2* mRNA, although they did not present RT–PCR data. We also carried out RT–PCR using osteosarcoma biopsy specimens and detected the PCR amplicon of *Notch1* in all specimens after 25-cycle reaction, although RT–PCR revealed that expression of *Notch1* was decreased in osteosarcoma human specimens compared with normal bone.

To explore how Notch pathway activation contributes to osteosarcoma growth, we attempted to block the Notch pathway. Treatment of osteosarcoma cells with GSI to block Notch activation prevented osteosarcoma growth *in vitro* and *in vivo*. Our findings suggested an association between activation of Notch signalling and pro-oncogenic effects in the progression of human osteosarcoma. Although GSIs inhibit the cleavage and activation of Notch receptors (Deftos *et al*, 2000), they neither specifically inhibit Notch receptors nor inhibit cleavage of a number of other transmembrane proteins (Esler *et al*, 2000; Zhang *et al*, 2000; Ni *et al*, 2001; Marambaud *et al*, 2002). When we used *CBF1* siRNA, a Notch cooperative transcriptional factor, siRNA also inhibited osteosarcoma growth effectively. These findings suggest that inhibition of Notch signalling prevents osteosarcoma growth. The role of GSIs in inhibiting the growth of osteosarcomas showed in this study is consistent with the findings of earlier studies of other malignancies (Pece *et al*, 2004; Curry *et al*, 2005; Park *et al*, 2008; Bin Hafeez *et al*, 2009; Meng *et al*, 2009; Real *et al*, 2009). Although oncoproteins are not usually tumorigenic when expressed alone, constitutively active NIC expression promotes papillary tumorigenesis (Klinakis *et al*, 2006). These findings showed that the Notch pathway is a strong activator of cell proliferation. Flow cytometry and ki67 staining showed that GSI promoted G1 arrest in osteosarcoma *in vitro* and inhibited tumour growth *in vivo*. We also found that GSI treatment regulated the expression of cell cycle regulators by Notch inhibition. Real-time PCR and western blot analysis revealed that cyclin D, E, SKP2, c-Myc, and pRB were downregulated, and p21^Cip1^ was upregulated upon Notch inhibition with GSI. Cyclins D, E, pRb, c-Myc, and SKP2 have been reported to promote G1-S phase progression (Swanton, 2004; Hwang and Clurman, 2005). It has been reported that cyclins D, E, and SKP2 are direct targets of Notch (Leong and Karsan, 2006). In addition, SKP2 has been reported to be a component of ubiquitin E3 ligase regulating G_1_/S transition by degradation of p21^cip1^ (Yu *et al*, 1998). p21^cip1^ can bind to various CDKs, including cyclin D/CDK4, cyclin E, and cyclin A/CDK2, and inhibits their kinase activity. Suppression of the c-Myc oncogene induces cellular senescence and tumour regression in osteosarcoma (Wu *et al*, 2007). Our findings suggest that Notch signalling has the same effect against osteosarcoma as in other previously studied cancers. Recently, Zhang *et al* (2008) found, using soft agar assay and xenografts, that downregulation of Notch signalling by compound E suppressed osteosarcoma cell invasion. On the other hand, downregulation of Notch signalling by GSI (compound E) had no effect on cell proliferation or tumorigenesis. One possible explanation for this discrepancy in findings may be differences in cell lines used or GSI. We also carried out soft agar assay, and found that GSI effectively inhibited colony formation in soft agar assay (data not shown). Unfortunately, we have not yet prepared metastatic tumour models using human osteosarcoma cell lines, and have not yet examined the inhibitory effects on metastasis of GSI and *CBF1* siRNA *in vivo*. Nonetheless, both of these studies provide independent support for the possibility that Notch pathway inhibition may be useful in treating osteosarcoma. It has been reported that GSI treatment induced apoptosis in Kaposi's sarcoma tumour cells (Curry *et al*, 2005). We carried out cell death detection assay, but could not detect the differences after GSI treatment in HOS and 143B HOS cell lines. These findings may have been due to differences in cell viability between osteosarcoma and Kaposi's sarcoma cell lines.

In conclusion, our findings show that the Notch signalling system is functionally activated in human osteosarcoma. This novel finding adds to the understanding of osteosarcoma and may be important in understanding the proliferation of osteosarcoma cells. Furthermore, the finding of growth inhibition by GSI, a Notch inhibitor, suggests that inactivation of Notch may be a useful approach to the treatment of patients with osteosarcoma.
