# Supplementary material for: Inhibition of Notch pathway prevents osteosarcoma growth by cell cycle regulation
Source: Br J Cancer. 2009 May 19;100(12):1957–65. doi: 10.1038/sj.bjc.6605060 (PMC2714252; doi:10.1038/sj.bjc.6605060)
Supplement: Supplementary Figures [file 6605060x1.ppt]

## Slide 1
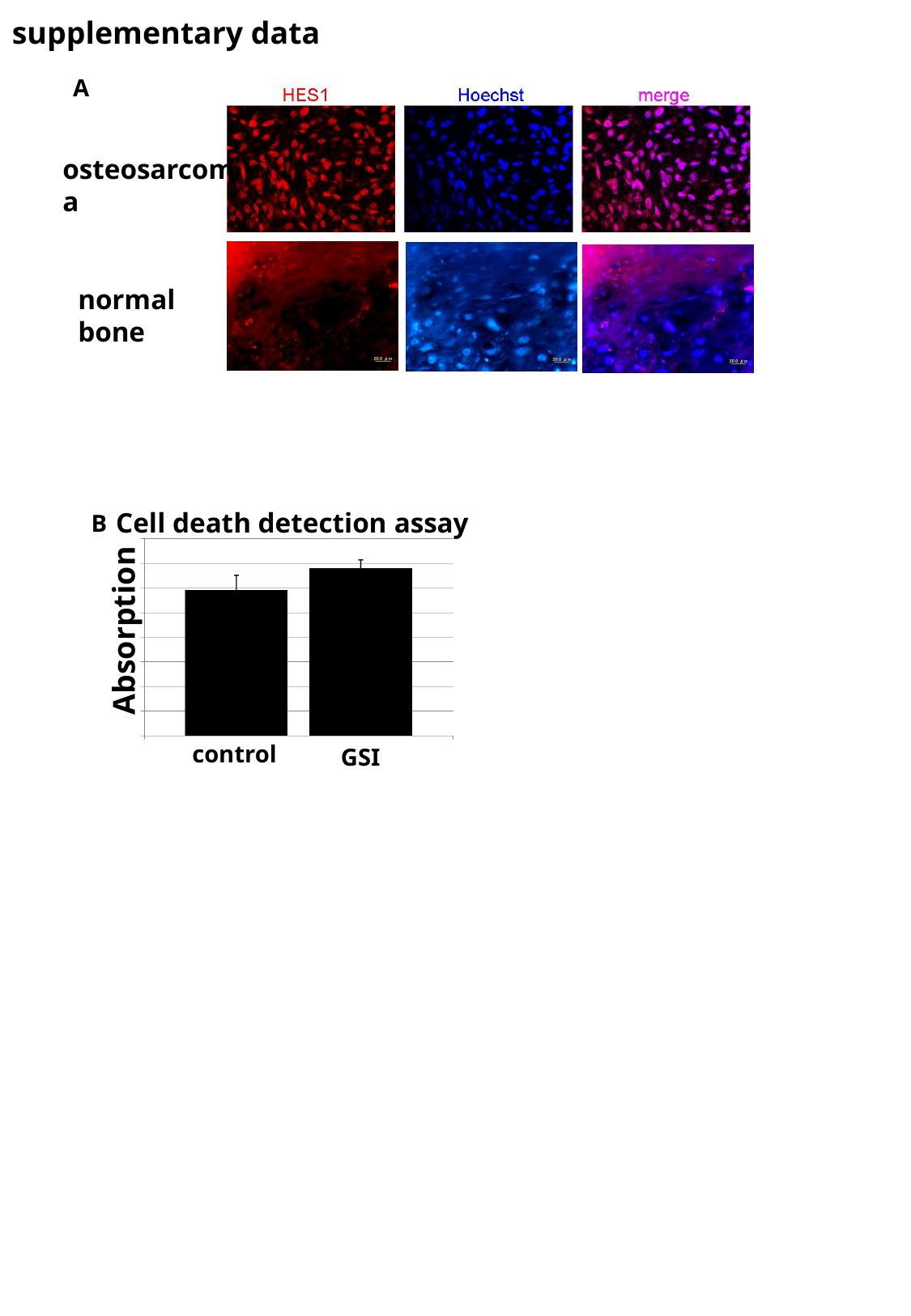

supplementary data
A
osteosarcoma
normal bone
Cell death detection assay
B
Absorption
control
GSI
