# Supplementary material for: Inhibition of Notch pathway prevents osteosarcoma growth by cell cycle regulation
Source: Br J Cancer. 2009 May 19;100(12):1957–65. doi: 10.1038/sj.bjc.6605060 (PMC2714252; doi:10.1038/sj.bjc.6605060)
Supplement: Supplementary Figures Legends [file 6605060x2.doc]

Supplementary data

Cell death detection assay revealed that GSI did not induced cell death (**A**).

Immunohistochemical examination revealed that HES1 was localized in the nucleus of osteosarcoma patient’ specimens (**A**).
